# Effect of Height on Perceived Exertion and Physiological Responses for Climbers of Differing Ability Levels

**DOI:** 10.3389/fpsyg.2020.00997

**Published:** 2020-06-05

**Authors:** Jan Gajdošík, Jiří Baláš, Nick Draper

**Affiliations:** ^1^Faculty of Physical Education and Sport, Charles University, Prague, Czechia; ^2^School of Health Sciences, College of Education, Health and Human Development, University of Canterbury, Christchurch, New Zealand

**Keywords:** sport climbing, energy cost, indirect calorimetry, treadwall, indoor climbing

## Abstract

**Purpose:**

The purpose of this study was to examine differences in perceived exertion (RPE) and physiological responses for climbers of different abilities completing an identical route low and high above the ground.

**Materials and Methods:**

Forty-two male (*N* = 18) and female (*N* = 24) sport climbers divided into three groups, lower-grade (*N* = 14), intermediate (*N* = 14), and advanced climbers (*N* = 14), completed two visits to a climbing gym, separated by 7 days. In a random order, the climbers completed a close-to-the-ground ascent (treadwall) and climb to height (climbing gym). Immediately after the test, climbers provided their RPE (6–20). Indirect calorimetry was used to assess physiological response during the ascent and recovery.

**Results:**

The mean (±standard deviation) RPE was higher for lower-grade climbers when ascending the route on the wall (RPE = 12 ± 1) when compared to the treadwall route (RPE = 11 ± 1, *P* = 0.040; *d* = 0.41). For all ability groups, the physiological response was higher on the climbing gym wall as opposed to the treadwall: ventilation (*P* = 0.003, η_*p*_^2^ = 0.199), heart rate (HR) (*P* = 0.005, η_*p*_^2^ = 0.189), energy cost (EC) (*P* = 0.000, η_*p*_^2^ = 0.501). The RPE demonstrated a moderate relationship with physiological variables (*R*^2^ = 0.14 to *R*^2^ = 0.45).

**Conclusion:**

Climbing to height induced a greater metabolic stress than climbing at a low height (treadwall) and led to higher RPE for lower-grade climbers. In this study, RPE appeared to be a good proxy measure of the physiological demands for advanced climbers but not for intermediate and lower-grade climbers. Therefore, using RPE in climbing with less experienced athletes may perhaps overestimate actual exercise intensity and should be interpreted carefully.

## Introduction

Sport climbing is a sport that can improve aerobic fitness and health (Rodio et al., 2008; Aras and Akalan, 2016). In recent years, indoor climbing has become more popular than rock climbing due to the increasing availability of indoor facilities such as indoor climbing gyms, bouldering walls, or treadwalls (Heil, 2019). Indoor climbing walls try to replicate outdoor rock climbing conditions, utilizing artificial holds and structures to create predefined routes typically of 15–25 m in height. In ascending a route at an indoor wall, climbers are exposed to physiological and psychological stress, according to the overall difficulty and climbing style used (Hodgson et al., 2009; Draper et al., 2010; Dickson et al., 2012; Fryer et al., 2013). In contrast, treadwalls, mechanical or motorized ergometers equipped with climbing holds, provide a physiological challenge where the risk of fall or fear from height is minimal. This type of ergometer enables the analysis of physiological responses to climbing in a controlled setting. Treadwalls can be altered to assess the effect of speed or inclination at submaximal or maximal intensity on physiological response in climbers (Watts and Drobish, 1998; España-Romero et al., 2009; Fryer et al., 2018; Limonta et al., 2018; Heil, 2019).

To date, research regarding the psychophysiological response to climbing suggests that fall potential increases somatic anxiety (autonomous hyperactivity and somatic tension such as breathlessness, cold sweat, and trembling), plasma cortisol, blood lactate and catecholamine concentrations, heart rate (HR), and oxygen cost and is associated with lower self-confidence for lower-grade and intermediate climbers but not for elite athletes (Hodgson et al., 2009; Draper et al., 2010; Dickson et al., 2012; Fryer et al., 2013; Baláš et al., 2017). Differences in stress response might not have resulted solely from the safety protocol (top-rope vs. lead climbing) but may simply result from the effect of height. When prescribing training programs or developing a research intervention, coaches, fitness instructors, and researchers should consider whether physiological responses are due to physical effort alone or the result of a combination of psychological and physiological factors. This is especially important when prescribing exercise intensity in health-oriented programs, as apparently high-intensity exercise (due to increased HR from psychological stress) may induce low or no muscle adaptation changes.

Subjective scales such as the rating of perceived exertion (RPE) are widely used instruments to assess exercise intensity and have been validated against several physiological outcomes (Chen et al., 2002). For instance, the American College of Sport Medicine (ACSM, 2014) guidelines use Borg’s scale of RPE along with HR and oxygen consumption for exercise intensity prescription. Although RPEs were initially created to score exercise intensity, it has been shown that RPE is affected additionally by psychological variables such mood state (anxiety, neurosis, and depression) or competitive strategy (Morgan, 1973; Robertson and Noble, 1997). When exercise intensity is low, the perception of effort is influenced primarily by non-physiological factors; when exercise intensity is high, physical demands mainly affect effort perception (Hall et al., 2005). Therefore, submaximal climbing from low to moderate intensity should induce differences in RPE if the same route is completed at height and close to the ground. We hypothesized that RPE and physiological response will differ for lower-grade climbers in the situation of stress from height, but not for intermediate and advanced climbers.

The purpose of this study was to compare RPE and physiological response in lower-grade, intermediate, and advanced climbers during climbing an identical route on the ground and ascending to height.

## Materials and Methods

### Participants

Forty-two male (*N* = 18) and female (*N* = 24) sport climbers participated in the study. Participants were divided into three groups, lower-grade (*N* = 14), intermediate (*N* = 14), and advanced climbers (*N* = 14), according to self-reported best red point grade in the last 3 months (Draper et al., 2016). Anthropometric and training characteristics are shown in Table 1. All subjects were asked to avoid intense exercise for 24 h prior to visits and to restrain from caffeine the day of testing. All participants gave written informed consent at the beginning of the study. The local university’s ethics committee granted approval for the study.

**TABLE 1:** Anthropometric and training characteristics (mean ± SD) in lower-grade, intermediate, and advanced female and male climbers.

Females	Lower grade (*N* = 12)	Intermediate (*N* = 8)	Advanced (*N* = 4)
Age (years)	31.6 ± 11.3	25.7 ± 4.3	31.3 ± 7.5
Body mass (kg)	62.6 ± 6.0	56.4 ± 7.1	53.5 ± 1.3
Height (cm)	168.1 ± 4.6	169.6 ± 7.2	162.8 ± 7.6
Climbing ability (IRCRA)	9.0 ± 1.3	13.5 ± 1.2	19.8 ± 1.3
Climbing experience (years)	9.7 ± 12.4	5.6 ± 3.8	10.0 ± 5.6

**Males**	**Lower grade (*N* = 2)**	**Intermediate (*N* = 6)**	**Advanced (*N* = 10)**

Age (years)	26.3 ± 4.4	29.6 ± 2.6	31.3 ± 6.5
Body mass (kg)	75.5 ± 6.4	74.2 ± 5.5	69.2 ± 5.5
Height (cm)	182 ± 5.7	183.3 ± 6.6	178.3 ± 8.1
Climbing ability (IRCRA)	9.5 ± 0.7	12.6 ± 0.6	19.2 ± 1.6
Climbing experience (years)	1.8 ± 0.4	3.1 ± 2.1	13 ± 5.2

### Study Design

All participants completed the routes on two separate visits on the climbing wall and with 7 days between each test. During a visit, they performed, in a randomly assigned order, a test on a treadwall (low over the ground) or on an indoor climbing wall (high over the ground). The testing started with standardized warm-up exercises (5 min running, 5 min mobilization exercises, and climbing low over the ground to learn the climbing sequence). Ten minutes of seated rest was provided to assess resting physiological response. Then, a climb of a 19.5-m-long route on the climbing wall and on the treadwall was completed at given speed (4 m⋅min^–1^). Immediately after completing the route, participants were asked to rate their exertion; then a further 10 min of seated rest was provided to assess excess postexercise oxygen consumption (EPOC).

### Climbing Routes

Treadwall and indoor wall routes both were vertical and have the same configuration of holds. Three identical sequences were repeated on the 19.5-m length and were graded 7 on the International Rock Climbing Research Association (IRCRA) scale. On the indoor wall route, climbers were belayed by an experienced instructor through a preinstalled rope (top-rope condition), and the risk of fall was minimal. To control the speed of ascent, the route was labeled with colored marks every meter. These marks had to be attained after 15 s; moreover, the instructor navigated the climbers acoustically. During treadwall climbing (ClimbStation generation 1, Forssa, Finland), participants completed the ascent without the need for safety equipment such as a harness or rope. During the treadwall ascent, climbers’ feet were maximally 0.5 m above the landing mat.

### Perceived Exertion and Physiological Response

Perceived exertion was assessed on a scale from 6 to 20 as suggested by Borg (1982). Immediately after the test, climbers were shown a table with numbers and corresponding verbal description of the exertion and indicated their exertion rating to the researcher.

Physiological responses were assessed using a breath-by-breath portable metabolic system (MetaMax 3B, Cortex Biophysik, Germany). The device was worn by climbers on the chest with a harness (total weight 1.4 kg). Gas calibration was performed using a reference gas (15% O_2_ and 5% CO_2_), and volume calibration was performed using a 3-L syringe. Breath-by-breath data were averaged at 20-s intervals and exported to Excel for further analysis.

Oxygen uptake (VO_2_), carbon dioxide production (VCO_2_), expiratory ventilation (V_*E*_), and breath frequency (BF) were measured by MetaMax 3B. Respiratory exchange ratio (RER) was computed by dividing VCO_2_ by VO_2_. EPOC was calculated from 10 min of sitting rest as total recovery VO_2_ minus resting VO_2_. The net climbing energy cost (EC) was computed from net climbing VO_2_ and EPOC using the energy equivalent for oxygen of 4.924 kcal. The chest belt (Polar Electro OY, Finland) was used for monitoring the HR, which was transmitted automatically to the MetaMax 3B.

### Statistical Analysis

Descriptive statistics (mean ± standard deviation) was used to characterize RPE and physiological response low and high over the ground in all ability groups. Differences between climbing conditions and ability groups were assessed by a 2 × 3 mixed model ANOVA with climbing route as the within-subject factor and ability as the between-subject factor. When significant, pairwise comparisons with Bonferroni corrections were applied. As unequal number of males and females completed the study across ability groups, the possible effect of sex was evaluated by ANCOVA with sex as the between-subject factor and climbing ability as the covariate. Statistical significance was set to *P* ≤ 0.05. Effect size was calculated using partial eta squared (η_*p*_^2^) and Cohen’s *d*, where values of 0.05, 0.10, and >0.20 represent small, intermediate, and large effects and 0.2, 0.5, and >0.8 represent small, moderate, and large differences for η_*p*_^2^ and Cohen’s *d*, respectively.

## Results

**FIGURE 1:**
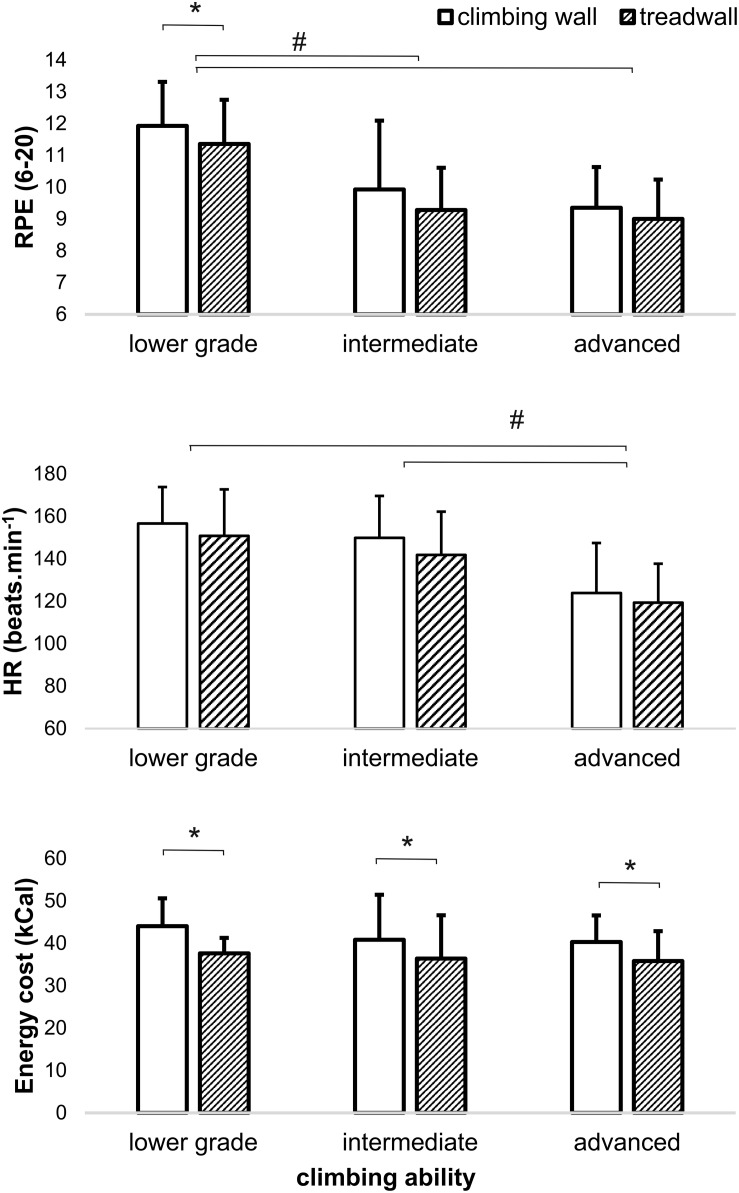
Mean (±SD) perceived exertion (RPE), heart rate (HR), and energy cost (EC) during climbing in height (climbing wall) and low (treadwall) to the ground. *Significant differences between climbing in height and low to the ground at *P* < 0.05. ^#^Significant differences between ability groups at *P* < 0.05.

Perceived exertions were higher when climbing to height as opposed to climbing low to the ground on the treadwall (+5.3%, *P* = 0.013, η_*p*_^2^ = 0.149). Pairwise comparisons revealed statistical differences only for lower-grade climbers (*P* = 0.040; *d* = 0.41) (Figure 1). The physiological response was higher for ascending to height in comparison to climbing low to the ground for V_*E*_ (+7.7%, *P* = 0.003, η_*p*_^2^ = 0.199), HR (+4.5%, *P* = 0.005, η_*p*_^2^ = 0.189), and EC (+14.0%, *P* = 0.000, η_*p*_^2^ = 0.501). However, pairwise comparisons indicated statistical differences in all ability groups only for EC: lower-grade climbers (*P* = 0.003, *d* = 1.26); intermediate climbers (*P* = 0.001, *d* = 0.43); and advanced climbers (*P* = 0.006, *d* = 0.67) (Figure 1 and Table 2).

**TABLE 2:** Mean (±SD) oxygen consumption (VO_2_), pulmonary ventilation (V_*E*_), breath frequency (BF), respiratory ratio (RER), and energy cost (EC) during climbing in height and low to the ground in lower-grade, intermediate, and advanced climbers.

	VO_2_ (ml⋅min^–1^⋅kg^–1^)	V_*E*_ (L⋅min^–1^)	BF (breaths⋅min^–1^)	RER	EC (kcal⋅kg^–1^)
lower grade_*treadwall*_	26.2 ± 2.6	48.8 ± 7.2**¤**	36 ± 6*¤	0.90 ± 0.06**¤**	0.59 ± 0.07*
lower grade_*indoor wall*_	26.4 ± 4.5	53.1 ± 11.2**¤**	37 ± 8*¤	0.93 ± 0.09^#^**¤**	0.69 ± 0.08*
intermediate_*treadwall*_	24.9 ± 4.6	43.3 ± 12.0	34 ± 8¤	0.86 ± 0.06¤	0.57 ± 0.09*
intermediate_*indoor wall*_	26.4 ± 3.6	45.0 ± 12.7	33 ± 9	0.86 ± 0.06^#^	0.64 ± 0.11*
advanced_*treadwall*_	24.6 ± 3.2	35.9 ± 6.4**¤**	24 ± 7¤**¤**	0.81 ± 0.04¤**¤**	0.56 ± 0.08*
advanced_*indoor wall*_	25.9 ± 2.3	39.8 ± 8.9**¤**	27 ± 4**¤**	0.83 ± 0.07**¤**	0.62 ± 0.06*

*Significant differences between climbing in height and low to the ground at *P* < 0.05. ^#^Significant differences between lower-grade and intermediate climbers at *P* < 0.05. ¤Significant differences between intermediate and advanced climbers at *P* < 0.05. **¤**Significant differences between lower-grade and advanced climbers at *P* < 0.05.

Lower-grade climbers perceived greater exertion than intermediate (*P* < 0.001, *d* = 1.29) and advanced climbers (*P* < 0.05, *d* = 1.86) for both climbing conditions. Moreover, lower-grade climbers demonstrated greater physiological response for BF (*P* = 0.001, *d* = 1.54), V_*E*_ (*P* = 0.002, *d* = 1.56), and HR (*P* < 0.001, *d* = 1.58) than advanced climbers (Figure 1 and Table 3). Additionally, intermediate climbers showed higher HR (*P* = 0.005; *d* = 1.18) than advanced climbers (Figure 1). No significant differences in EC between ability groups were stated.

**TABLE 3:** Relationship between perceived exertion (RPE) and mean oxygen consumption (VO_2_), pulmonary ventilation (V_*E*_), heart rate (HR), breath frequency (BF), respiratory ratio (RER), and energy cost (EC) during climbing in height and low to the ground.

	VO_2_	V_*E*_	HR	BF	RER	EC
RPE_*treadwall*_	0.287	0.481*	0.542*	0.377*	0.490*	0.245
RPE_*indoor wall*_	0.047	0.439*	0.414*	0.669*	0.627*	0.380*

*Significant relationship at *P* < 0.05.

No interaction of climbing ability and climbing condition was found. RPE and physiological variables demonstrated a moderate relationship (*R*^2^ = 0.14–0.45; Table 3).

Due to an uneven distribution of males and females in ability groups, sex comparison is presented in Table 4. Estimated marginal means did not show any difference between males and females for RPE and physiological variables except for BF.

**TABLE 4:** Differences between males and females in perceived exertion (RPE), heart rate (HR), oxygen consumption (VO_2_), pulmonary ventilation (V_*E*_), breath frequency (BF), and energy cost (EC).

	Males	Females	*P*	η _*p*_^2^
RPE (6–20)	10.0 ± 0.4	10.2 ± 0.3	0.706	0.004
HR (beats⋅min^–1^)	133 ± 5	145 ± 4	0.054	0.092
VO_2_ (ml⋅min^–1^⋅kg^–1^)	26.5 ± 0.8	25.2 ± 0.6	0.231	0.037
V_*E*_ (L⋅min^–1^)	47.9 ± 2.2	41.9 ± 1.8	0.052	0.093
BF (breaths⋅min^–1^)	28.8 ± 1.6	33.5 ± 1.3	0.038*	0.106
EC (kcal⋅kg^–1^)	0.62 ± 0.2	0.61 ± 0.2	0.786	0.002

Data are presented as estimated marginal means (±standard error) from treadwall and indoor wall climb with a correction for a common covariate (climbing ability = 13.8 IRCRA scale). *Significant differences at *P* < 0.05.

## Discussion

The aim of our study was to compare RPE and physiological demands to ascent identical routes low and high over the ground for climbers of differing ability. Climbing to height induced higher RPE than climbing low over the ground in lower-grade climbers. The differences in RPE were not repeated for higher-ability climbers. Moreover, RPE was only moderately related to physiological responses and consequently may not be a good indicator of physiological demands in climbing.

The results confirmed our hypothesis that height represents an important stress factor in climbing even in a top-roping condition where the risk of a fall is minimal. Interestingly, RPE when ascending to height was only elevated for lower-grade climbers, although metabolic stress was increased in all ability groups. Furthermore, results of Pearson product–moment correlations revealed only a moderate relationship between RPE and physiological variables, which means that factors other than mental stress induced elevated metabolic response when climbing to height or that the use of RPE in this situation was not specific or accurate enough. Elevated metabolic responses may be partially due to wearing a harness, the weight of which ranged from 250 to 600 g. Different movement patterns and different work/relief ratio on the treadwall and indoor wall might have also influenced the physiological response as suggested previously (Donath et al., 2013; Fryer et al., 2013). However, movement analysis was not performed due to the relatively large number of participants.

Perceived exertions during climbing in both conditions were moderately related to HR (*R*^2^ = 0.17–0.29). Lower-grade and advanced climbers rated both climbs as fairly light and very light on the RPE scale (∼12 and 9) which corresponded to HRs of ∼154 and 122 beats⋅min^–1^ and VO_2_ of ∼26 and 25 ml⋅min^–1^⋅kg^–1^, respectively. In a study by Scherr et al. (2013) with a large cohort of participants, an equation for HR estimate from RPE was proposed: HR (beats⋅min^–1^) = 69.34 + 6.23 × RPE (*R*^2^ = 0.55), which would correspond to values of 144 and 125 beats⋅min^–1^ for fairly light and very light RPEs in the current study. This estimate is valid for advanced climbers; however, lower-grade climbers demonstrated greater HR with respect to the prediction formula. HR was greater by ∼32 beats⋅min^–1^ in lower-grade climbers compared to advanced climbers while VO_2_ was elevated only by 1 ml⋅min^–1^⋅kg^–1^. This disproportionate rise of HR to VO_2_ was described by Mermier et al. (1997) and Sheel (2004) when climbing easy and more difficult routes and was explained by handgrip isometric contractions, arm position over the head, and psychological stress. Additionally, this disproportion was more elevated with a more intense handgrip contraction (Fryer et al., 2013). Therefore, to estimate subjectively physiological measures during climbing, RPE may be a valid tool in advanced climbers on easy climbs, irrespective of height of the ascent. However, in lower-grade and intermediate climbers or on more difficult ascent, RPE underestimates HR response.

In our study, climbing to height on an indoor climbing wall was metabolically more demanding than climbing low over the ground. The largest differences between the two conditions occurred for lower-grade climbers (Δ 10 kcal⋅kg^–1^) and the lowest for advanced climbers (Δ 6 kcal⋅kg^–1^). Lower-grade climbers were also the only group where significant differences in RPE were revealed. It is possible that the differences in EC in the two climbing conditions were due to a combination of psychological and technical factors. As this ability group has the lowest experience with the sport, climbing to height might have presented a more mentally demanding condition resulting in changes in the use of forces on hold during ascent. Previously, it has been demonstrated that less experienced climbers disproportionally load their arms than their legs, thereby increasing their physiological response when compared with higher-grade climbers (Baláš et al., 2014). In agreement with this difference, RPE has been found to be higher during arm exercise than with leg exercise (G. Pandolf et al., 1984; Borg et al., 1987).

Interestingly, differences in VO_2_ between ability groups or climbing conditions did not reach significance as expected (Bertuzzi et al., 2007). A possible explanation for between-group VO_2_ similarity might be related to the higher proportion of females in the lower-grade group and males in the advanced group. In previous research, female climbers have demonstrated more economical movement and, therefore, lower VO_2_ for the same climbing task (Heil, 2019). The intraindividual variation in VO_2_ between climbing low and high to the ground did not reach significance either. However, when including EPOC in calculation, we found significant differences in EC, which was greater after climbing to height, and this might be related to more pronounced anaerobic isometric contractions and/or greater catecholamine efflux.

The effect of sex on RPE and physiological variables was assessed by ANCOVA with control for climbing ability level. Females may have a different psychological approach than males to the climb, and it was, for example, discussed that males and females may rate physical exertion differently (Robertson and Noble, 1997). Our results did not show any differences between males and females for RPE, EC, and VO_2_; however, BF was significantly higher in females, and HR and V_*E*_ were close to significance level. V_*E*_ should be higher in males as they have larger body mass; however, HR and BF are not influenced by body shape. We acknowledge that some sex differences in stress responses may have been presented but were not detected by RPE. For future studies, design including only males and then females should be conducted to assess the effect of stress conditions on RPE.

Some other limitations have to be acknowledged. The route was climbed at one given speed in vertical profile. Climbing at a range of speeds on walls of altered inclinations might have provided different results. Climbers were divided into three ability groups, but the ratio of female and male climbers in these groups was not even. This might have led to bias for between-group comparisons. Nevertheless, the main purpose was to examine intraindividual differences, and the relatively large sample size and controlled settings will likely have increased the internal validity of the research.

## Conclusion

Climbing height induced greater metabolic stress than climbing low to the ground and, moreover, led to higher RPE in lower-grade climbers. The differences in RPE were not seen in higher-ability-level groups. RPE was a good indicator of physiological demands in advanced climbers on easy routes. With increasing difficulty or in lower-grade and intermediate climbers, RPE underestimated HR response.

## Data Availability Statement

The datasets generated for this study are available on request to the corresponding author.

## Ethics Statement

The studies involving human participants were reviewed and approved by the Ethics Committee of the Faculty of Physical Education and Sport, Charles University. The patients/participants provided their written informed consent to participate in this study.

## Author Contributions

JG developed the theoretical framework, conceived the study, collected the data, analyzed the data, and wrote the manuscript. JB developed the theoretical framework, conceived the study, analyzed the data, guided the analyses, and provided critical feedback on the drafts. ND provided critical feedback on drafts and edited the final manuscript for submission.

## Conflict of Interest

The authors declare that the research was conducted in the absence of any commercial or financial relationships that could be construed as a potential conflict of interest.
